# Diversity and Assemblage of Harmful Algae in Homestead Fish Ponds in a Tropical Coastal Area

**DOI:** 10.3390/biology11091335

**Published:** 2022-09-09

**Authors:** Liza Akter, Md. Akram Ullah, Mohammad Belal Hossain, Anu Rani Karmaker, Md. Solaiman Hossain, Mohammed Fahad Albeshr, Takaomi Arai

**Affiliations:** 1 Department of Fisheries and Marine Science, Noakhali Science and Technology University, Noakhali 3814, Bangladesh; 2 Environmental and Life Sciences Programme, Faculty of Science, Universiti Brunei Darussalam, Jalan Tungku Link, Gadong BE 1410, Brunei; 3 School of Engineering and Built Environment, Griffith University, Brisbane, QLD 4111, Australia; 4 Department of Oceanography, Shahjalal University of Science and Technology, Sylhet 3114, Bangladesh; 5 Department of Marine Science, Faculty of Science, Chulalongkorn University, Bangkok 10330, Thailand; 6 Department of Zoology, College of Science, King Saud University, P.O. Box 2455, Riyadh 11451, Saudi Arabia

**Keywords:** diversity, harmful algae, environmental variables, homestead ponds

## Abstract

**Simple Summary:**

Harmful algae are those which release toxins to the aquatic ecosystems. Excessive growth of these algae can kill fish, create anoxia, impede aquaculture activities and contaminate aquatic food. Therefore, it is important to investigate their occurrence, diversity and abundance in pond aquaculture systems. In this study, we have identified 81 genera of harmful algae from 30 coastal homestead ponds mainly consisting of *Microcystis* spp. (30.14%) and *Actinoptycus* spp. (18.32%). Based on taxonomic classes, the community assemblage was dominated by Cyanophyceae, Chlorophyceae and Bacillariophyceae. Statistical analyses demonstrated that that dissolved oxygen, nitrates, phosphates, sulphates, salinity and transparency influence the abundance of identified algal genera.

**Abstract:**

Algae are the naturally produced food for fish in any aquatic ecosystem and an indicator of a productive pond. However, excess abundance of harmful algae can have detrimental effects on fish health. In this study, the algal communities of 30 coastal homestead fish ponds were investigated to identify the diversity, assemblage and controlling environmental variables of harmful algae from a tropical coastal area. The findings showed that 81 of the 89 genera of identified algae were harmful, with the majority of them being in the classes of Cyanophyceae (50.81%), Chlorophyceae (23.75%), Bacillariophyceae (9.5%), and Euglenophyceae (8.47%). *Microcystis* spp. alone contributed 28.24% to the total abundance of harmful algae. Significant differences (*p* < 0.05) in algal abundance were found among the ponds with the highest abundance (470 ± 141.74 × 10^3^ cells L^−1^) at pond (S_25_) near agricultural fields and the lowest abundance (109.33 ± 46.91 × 10^3^ cells L^−1^) at pond (S_14_) which was lacking sufficient sunlight and nutrients. Diversity indices, e.g., dominance (D), evenness (J′), richness (d) and Shannon diversity index (H′) ranged from 0.17 to 0.44, 0.23 to 0.6, 0.35 to 2.23 and 0.7 to 1.79, respectively, indicating a moderate range of diversity and community stability. Community composition analysis showed the assemblage was dominated by Cyanophyceae, Chlorophyceae and Bacillariophyceae, whereas, multivariate cluster analyses (CA) identified 11 major clusters. To identify the factors controlling their distribution or community assemblages, eight environmental variables (temperature, pH, dissolved oxygen (DO), salinity, transparency, nitrates, phosphates and sulphate) were measured. ANOVA analysis showed that the variables significantly differed (*p* < 0.05) among the ponds, and canonical correspondence analysis (CCA) demonstrated that DO, nitrates, phosphates, sulphates, salinity and transparency have the most impact on the abundance of algal genera. In addition, analyses with Pearson’s correlation coefficient showed that the abundance of total algae, diversity and community were mainly governed by phosphates and sulphates. These results can be used to identify and control these toxic algal groups in the local aquaculture sector.

## 1. Introduction

Algae are the best index of the biological productivity of aquatic habitats [1]. They are the primary food producers of any aquatic ecosystem [2,3] and play a significant role in stabilizing the pond ecosystem. The algae are very efficient at absorbing carbon dioxide and absorb almost 50% of carbon dioxide on earth. Like phytoplankton, algae are also a positive element in the ecological chain. However, in addition to these positive roles of algae in aquatic ecosystems, some of them can be harmful to animals and humans as they release toxins [4]. When these harmful algae grow quickly and accumulate in an area of water, the phenomenon is known as a harmful algal bloom (HAB). HABs are recognised as a major environmental problems in many countries. In particular, Cyanobacterial species are more harmful for freshwater ecosystems as they produce more toxic substances which are harmful for the human body and aquatic organisms. Cyanobacterial toxicity is very common in the fresh water ponds of tropical areas including Bangladesh. Only a concentration of 1 µg L^−1^ of *Microcystis* sp., which is extremely hazardous, can contain hepatotoxins, according to WHO guidelines. However, the mean density of *Microcystis* sp. and a group of cyclic hepatotoxins produced by cyanobacterial species often exceeds the preliminary WHO recommendation in Bangladesh (>10 µg L^−1^). Physical-chemical factors appear to be crucial in stabilising the succession of phytoplankton communities and the subsequent diversity by favouring or restricting the growth of various phytoplankton groups [5,6].

Plankton distribution, abundance and diversity indicate the health condition of an aquatic environment [7]. Monitoring systems for plankton are crucial because they provide information about their appearance in systems. They are a warning system for potentially dangerous blooms, and they may even identify predictive variables. One effective way to identify and assess the effects of pollution on aquatic populations is through the use of species diversity indices in combination with physical and chemical factors [8]. To understand community structure and assess water quality, diversity indices of algae are required [9,10,11,12].

In aquatic ecosystems, physical-chemical characteristics are crucial in determining the succession, variety, and community of algae and are also important factors in controlling the growth of algae [5,6]. Sunlight, temperature, daylight duration, transparency, and nutrients such as nitrogen and phosphorus are the physico-chemical variables that have the most impact [13]. Long sunshine hours induce algae to develop more quickly [14], but low water clarity causes growth to slow down [15]. Nitrates are the most significant nutrient that algae directly consume for growth, with phosphate coming in second [16].

A combination of favourable environmental factors in a freshwater habitat might result in an algal bloom. Among them, nutrients, light, temperature, and stable water column conditions are the key controlling variables. The main sources of nutrients in freshwater bodies are sewage, household waste, runoff, and flushes from agricultural lands. Algal growth is promoted by eutrophication, or an increase in nutrients. The blooms typically appear during the warmer seasons of the year, or when the water is warmer and the amount of light is higher. The ideal temperature for the growth of blue-green algae is about 25 °C. Algal growth is also accelerated by thermal stratification in a body of still water. Summertime thermal stratification with two layers of water—warm and cold—occurs when there is no water movement.

In the coastal area of Bangladesh, almost every house possesses a small pond adjacent to their house. There are about 4 million homestead owned ponds for aquaculture throughout Bangladesh, covering an area of 266,259 ha in the neighborhood of households [17]. Noakhali is a southern central coastal belt in Bangladesh which is very rich in homestead ponds. About 3 to 15% of household income and 25 to 50% of all fish consumption can come from these homestead ponds [17]. The ponds in this area are used for a variety of domestic functions such bathing, washing, cleaning, watering animals, and occasionally for providing drinking water as well as fish culture [18]. Extensive fish culture is typically carried out in these types of ponds using only natural foods, such as phytoplankton and zooplankton [19]. Because of increasing natural food production farmers can use locally available fertilizer (e.g., cow-dung) which results in high nutrient levels in the water body. As a result, sometimes more harmful algae than useful species occur in the water body.

Numerous studies have been conducted on the phytoplankton or algal diversity of fresh water aquaculture ponds in Bangladesh, including those on the physico-chemical parameters and phytoplankton abundance of homestead ponds in Noakhali [20]; plankton distribution in fish ponds of Noakhali [21]; physico-chemical parameters and phytoplankton communities of aquaculture ponds [22]; plankton in two culture ponds [19]; the diversity of phytoplankton in seasonal waterlogged paddy fields [23]; and phytoplankton and cyanobacteria in earthen fish ponds from Khulna, Rajshahi and Mymensingh [24,25,26,27]. However, none of these studies have investigated the harmful algal diversity in freshwater aquaculture ponds in Bangladesh. In addition, there is no data on the physico-chemical parameters, harmful algal abundance and species composition of homestead ponds from Noakhali, Bangladesh. Considering this knowledge gap, and the useful and harmful impacts of algae on fish culture, this study was designed to identify the diversity, assemblage and controlling environmental variables of harmful algae in the homestead ponds of the central coastal area of Bangladesh.

## 2. Materials and Methods

### 2.1. Study Site and Sampling Design

Generally, the urban homestead ponds of Noakhali are very small in size as they are formed through mining of soil to build houses or save houses form floods. Bangladesh is a river-based country and for that reason almost every year the country faces floods. The homestead ponds are used for various purposes, such as drinking water, bathing, washing, watering and bathing cattle, and sometimes used for irrigation of cultivated fields.

**Figure 1:**
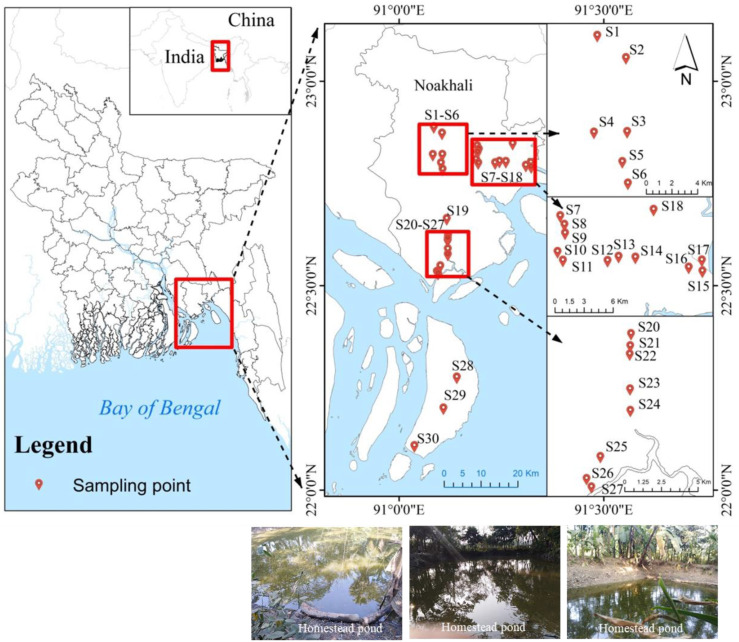
Location map showing the selected sampling points (S_1_, S_2_, S_3_, S_4_, S_5_, S_6_—Noakhali; S_7_, S_8_, S_9_, S_10_, S_11_, S_12_—Kabirhat; S_13_, S_14_, S_15_, S_16_, S_17_, S_18_—Companiganj; S_19_, S_20_, S_21_, S_22_, S_23_, S_24_—Subarnachar; S_25_, S_26_, S_27_, S_28_, S_29_, S_30_—Hatiya).

For this research, five central coastal sub-districts of Noakhali were selected: 1. Noakhali; 2. Khabirhat; 3. Companiganj; 4. Subarnachar; and 5. Hatiya. From each upazila (administrative area), six homestead ponds where algal blooms had formed with minimum or maximum level were selected randomly while maintaining a minimum of 1km distance between the selected ponds. Finally, 30 ponds (S_1_–S_30_) were selected between latitudes 22°00′ and 23°00′ N and longitudes 91°00′ and 91°30′ E in southern Bangladesh (Figure 1).

Three replicate samples were taken, and the mean values were calculated for the algal community and physico-chemical parameters. The water samples were collected between November 2020 and January 2021 from 8: 00 a.m. to 4: 00 p.m.

### 2.2. Water Sample Collection, Identification and Enumeration of Algae

A 100 mL concentrate sample was taken from 10 litres of surface water using a 63-µm meshed phytoplankton net, and three replicate samples from each pond were taken and placed in three different transparent plastic bottles. For further examination, 100 mL of each bottle’s sample was stored with a 5% buffered formalin solution. With the use of standard manuals, textbooks, and research articles, the algae were identified to the genus level utilising a light microscope (Carl Zeiss Axiostar microscope and Euromax, EC 1152 microscope) [28,29,30,31,32]. Density measurements of algae were performed using a Sedgwick Rafter cell and the abundance was expressed as cells L^−1^.

For algal identification and counting, a 1ml sample was placed in a Sedgwick-Rafter (S-R) cell and left to settle for 5 min. Then 10 random S-R cells were counted and the density of algae was calculated by using the following formula and expressed as cell L^−1^ [33]:

$$N=\frac{P\times C\;\times 100}{L}$$

where N = the total number of algae per liter of sample water, P = the average number of algae counted from 1 mL of water sample, C = the volume of the concentrated water sample (100 mL), and L = the total volume of the water which was used for concentrating the water sample (10 L).

### 2.3. Diversity Indices

The following formula was used to calculate the Dominance Index (D) [10], Species Richness (d) [12], Species Evenness (J) [34], and Shannon–Wiener Index (H′) [35]:

$$D=\overset{s}{\underset{i=1}{\sum }}\left(\mathrm{ni}///N\right)2$$

$$d=\frac{S-1}{\ln\left(N\right)}$$

$$J^{\prime }=\frac{H^{\prime }}{\ln\left(S\right)\;}$$

$$H^{\prime }=-\sum_{i=1}^{S}\mathrm{Pi}\;(\ln\mathrm{Pi})\;$$

where Ni = number of individuals of the i^th^ species; S = total number of species; Pi = ni/N for the i^th^ species; ni = number of individuals of a species in sample and N = total number of individuals.

### 2.4. Physico-Chemical Parameters

Hannah multi-parameters (Model: H198194) were used to monitor the physico-chemical parameters such as water temperature (°C), pH, dissolved oxygen (mg L^−1^), and salinity (ppm). Water transparency (in cm) was measured using a Secchi disc. Measurements of nitrate (mg L^−1^), phosphate (mg L^−1^), and sulphate (mg L^−1^) were made using a spectrophotometer (Model: DR 2700). For the analyses of nutrient levels, 150 mL of filtered water was collected and kept in a cooled icebox in the field [9] and stored in refrigerator for later analysis by standard methods [36].

### 2.5. Statistical Analysis of Collected Data

For the community and diversity analyses, both uni-and multivariate statistical analyses were conducted. Normality and homoscedasticity were examined prior to data analysis. One-way ANOVA tests were used to examine the variation in physico-chemical parameters (temperature, pH, DO, salinity, transparency), nutrients (nitrates, phosphates, sulphates) at a 5% significant level. The Kruskal–Wallis ANOVA was used to test for significant differences in different algae classes, total algae abundance, abundance of dominant genera, algae species richness, evenness of algae species, and the Shannon–Wiener values for algae species among different stations for non-normal and heteroscedastic data (biological variables, where different groups have different standard deviations). The Bray–Curtis similarity measure of the dominant genera was used to conduct the cluster analysis. To ascertain the association between the dominant algae genus and physico-chemical parameters, as well as to identify the important variables that regulate the distribution and abundance of the dominant algae genera, canonical correspondence analysis (CCA) was used. The associations between various physico-chemical properties, various algae assemblages, and diversity indices of algae were examined using the Pearson’s correlation coefficient. All of the univariate and multivariate analyses were performed using the programme PAST V3 (Palaeontological Statistics) [37].

### 2.6. Ethical Statement

No ethical issues were applicable in this research.

## 3. Results and Discussion

### 3.1. Algae Community and Composition

**Figure 2:**
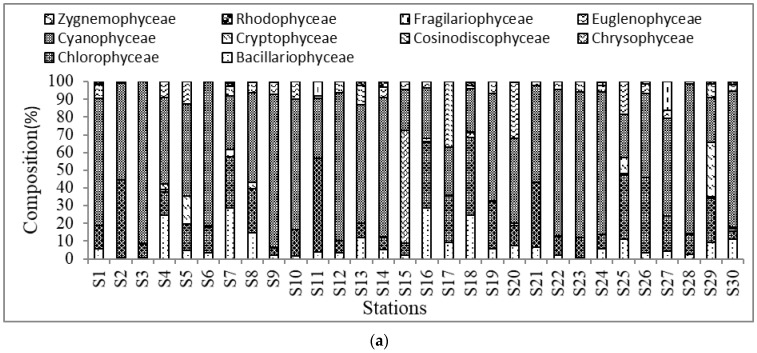

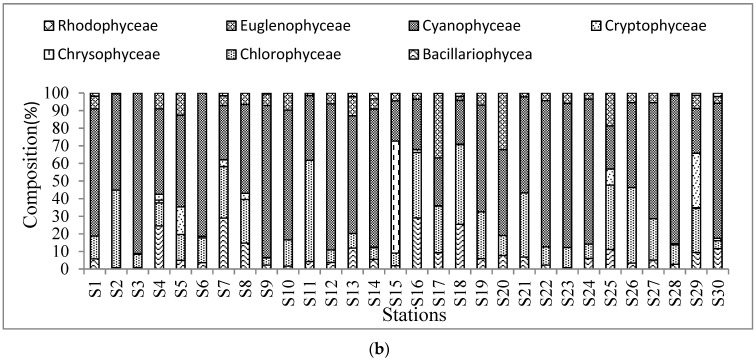
Composition (%) of (**a**) algal classes at different stations/ponds, (**b**) harmful algal classes at different stations/ponds.

A total of 89 genera of algae were identified from 10 major algal classes, namely, Bacillariophyceae (20 genera), Chlorophyceae (32 genera), Chrysophyceae (2 genera), Cosinodiscophyceae (2 genera), Cryptophyceae (1 genera), Cyanophyceae (21 genera), Euglenophyceae (4 genera), Fragilariophyceae (2 genera), and Rhodophyceae (1 genera), Zygnemophyceae (4 genera) (Table 1). The algal communities were mainly composed of Bacillariophyceae (9.36%), Chlorophyceae (23.55%), Chrysophyceae (3.81%), Cosinodiscophyceae (0.28%), Cryptophyceae (3.21%), Cyanophyceae (50.12%), Euglenophyceae (8.37%), Fragilariophyceae (0.86%), Rhodophyceae (0.36%) and Zygnemophyceae (0.08%) (Figure 2a,b). The highest percentage of Bacillariophyceae (28.85%) was recorded at S_16_ and the lowest (0.71%) was at S_23_, whereas, for the Chlorophyceae the highest (43.91%) was found at S_2_ and the lowest (4.04%) at S_9_. Most of the genera and classes recorded in this study were also reported in earlier studies [20,21,22]. However, we recorded higher numbers of genera than other studies [20,38], and the algal community and composition were also different, possibly due to geographical locations, seasonal blooms in water and variations in the physico-chemical parameters [39], as it is known that temperature, pH, DO and nutrients are the growth parameters mainly responsible for algal abundance [40,41]. The highest abundance (470 ± 141.74 × 103 cell L^−1^) of algae was recorded from the ponds near agriculture lands using organic and inorganic fertilizers, and the lowest abundance (109.33 ± 46.91 × 103 cell L^−1^) of algae was observed in some ponds which were lacking sunshine and nutrients. This trend was also observed by some other investigators from aquaculture ponds [16,20,22,25,27,42,43].

A total of 29 genera were considered as dominant based on their 1 × 10^3^ cell L^−1^ abundance, which was inconsistent with an earlier report [20] in which only 16 dominant genera were reported. However, some of the dominant genera were similar to their findings [20]. *Aphanocapsa* sp., *Microcystis* spp., *Chlorella* spp., *Heamatococcus* spp., *Oocystis* spp., *Phacus* spp., *Euglena* spp., *Traclomonus* sp., *Cosinodiscus* spp. were considered as dominant in this study, which agrees with the findings of Sarker et.al. [20]. *Phacus* spp., *Euglena* spp. were found as dominant in freshwater ponds, which agrees with the findings of some earlier reports [19,22,24,25,27,43].

The overall harmful algal community was composed of Bacillariophyceae (9.5%), Chlorophyceae (23.75%), Chrysophyceae (3.86%), Cryptophyceae (3.25%), Cyanophyceae (50.81%), Euglenophyceae (8.47%), and Rhodophyceae (0.36%) (Figure 2b). Out of 89 genera, 29 genera were recorded as dominant genera and most of them were included in harmful algae classes. The composition of all recorded dominant genera was as follows: *Actinoptycus* spp. (18.32%), *Aphanocapsa* sp. (0.66%), *Asterococcus* sp. (0.61%), *Aulocodiscus* sp. (0.44%)*, Botryococcus* sp. (0.3%), *Chlrococcus* sp. (1.28%), *Chlorella* spp.(18.79%), *Cladophora* sp. (0.51%), *Cloasterium* spp. (0.19%), *Coelastrum* spp. (0.71%), *Cosinodiscus* spp. (3.67%), *Euglena* spp. (2.04%), *Gomphospheria* spp. (1.9%), *Heamatococcus* spp. (1.32%), *Lemena* sp. (0.38%), *Lepocinclis* spp. (1.72%), *Microcystis* spp.(30.14%), *Navicula* spp. (0.57%), *Nitzschia* sp. (5.68%), *Oocystis* spp. (0.13%), *Pediastrum* sp. (0.38%), *Phacus* spp. (2.73%), *Phormidium* sp.(0.11%), *Planktospheria* sp. (1.59%), *Rivularia* sp. (0.63%), *Rhodomonus* spp. (3.08%), *Selenastrum* spp. (0.86%), *Stephanodiscus* sp. (0.29%), *Traclomonus* sp. (0.97%).

The density of harmful dominant genera varied from 439.67 ± 120.62 × 10^3^ cells L^−1^ to 99.67 ± 47.55 × 10^3^ cells L^−1^. It has been established that the algal growth is considered to be blooming if the density exceeds the concentrations of 1000 cells/mL [43]. This very large algal growth observed in the study area clearly indicates bloom formation. This type of bloom is often referred as a harmful algal bloom (HAB). HABs causes water discoloration and spread obnoxious smells. Through the generation of natural poisons, mechanical harm to other creatures, or other mechanisms, HABs harm other organisms. Numerous types of shellfish poisonings have been linked to HABs, which are frequently linked to mass mortality of fishes.

### 3.2. Algal Community Abundance and Diversity Indices

**Figure 3:**
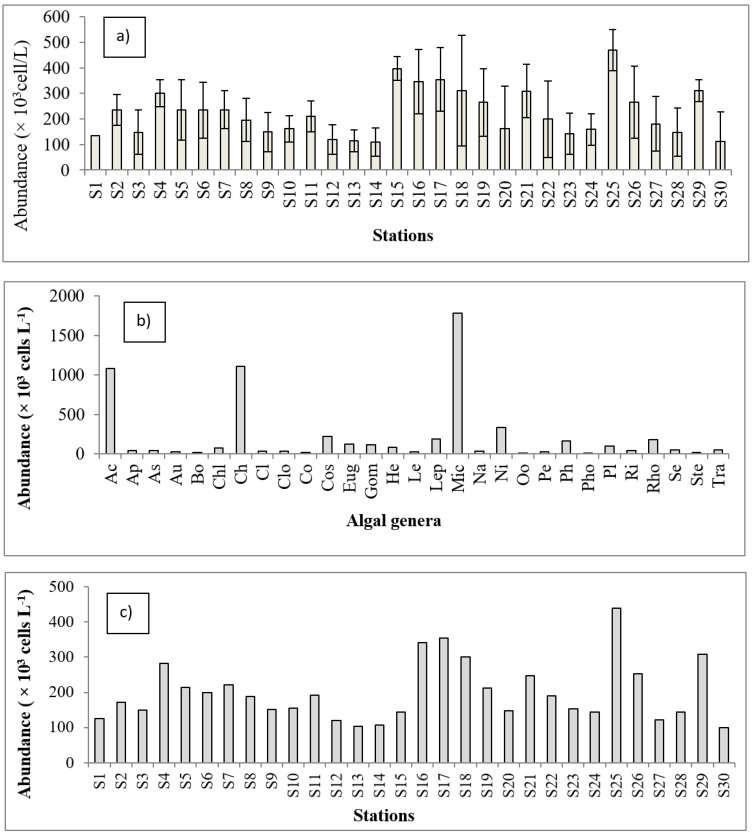

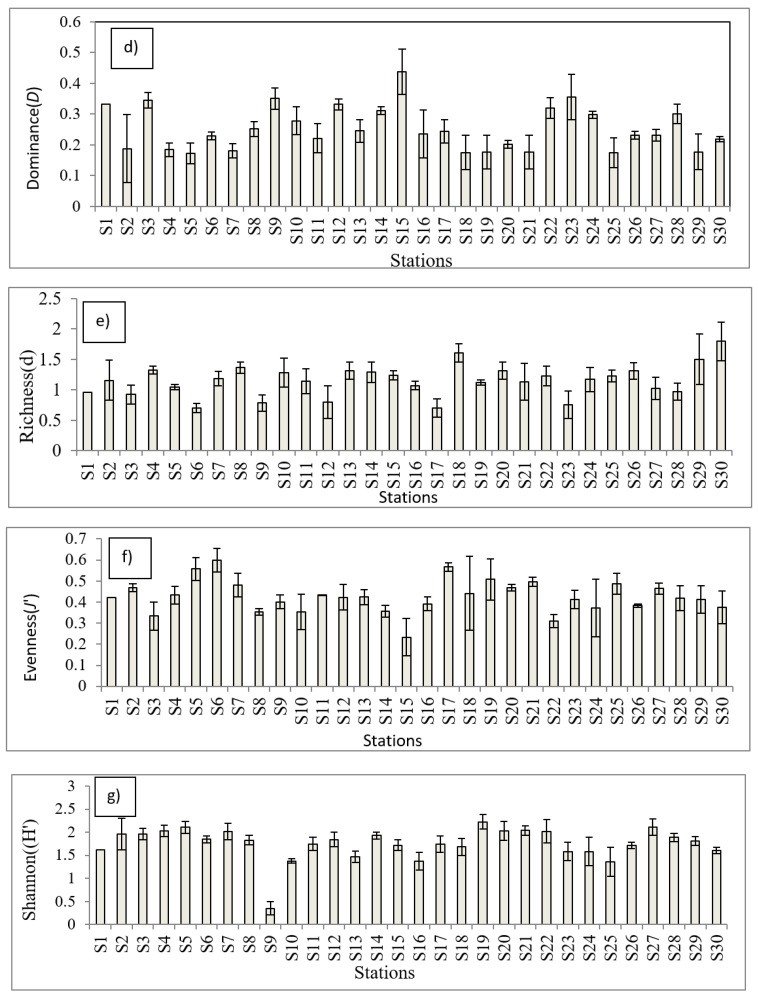
Abundance and diversity indices of algal communities: (**a**) Total mean abundance of algae, (**b**) the abundance of dominant genera, (**c**) the abundance of harmful dominant algal genera, (**d**) dominance index (D) of algae at different stations, (**e**) species richness of algae, (**f**) species evenness (J′) of algae, and (**g**) Shannon–Wiener diversity index (H′) of algae at different stations.

The highest abundance (470 ± 141.74 × 10^3^ cells L^−1^) of algae was found at S_25_ and the lowest abundance (109.33 ± 46.91 × 10^3^ cells L^−1^) was at S_14_ (Figure 3a). A one-way ANOVA showed a significant difference among different stations/ponds (*H* = 78.37, *p* = 1.993E−06). Among all the 29 dominant genera, *Microcystis* spp. was most abundant with 1780.05 ± 402.33 × 10^3^ Cells L^−1^ and the *Phormidium* sp. had the lowest abundance with a density of 6.34 ± 9.66 × 10^3^ cells L^−1^ (Figure 3b). Highly significant differences were found among the dominant genera (*H* = 54.76, *p* = 0.001816) in the studied ponds. The highest value (439.67 ± 120.62 × 10^3^ cells L^−1^) of harmful dominant genera was found at S_25_ and the lowest value (99.67± 47.55 × 10^3^ cells L^−1^) was recorded from S_30_ (Figure 3c). The ANOVA analysis detected no significant difference in the mean values of algal abundance for the dominant taxa among stations (*H* = 21.02, *p* = 0.8584).

The highest dominance index value (0.44 ± 0.08) was found at S_15_ and the lowest value (0.17 ± 0.01) at S_5_ (Figure 3d). The ANOVA analysis showed highly significant differences in the dominance index value at different stations (*H* = 71.34, *p* = 1.982E−05). This value indicates the low dominancy of algae [10] in the homestead ponds which was consistent with earlier findings [20,44]. The species richness varied from 1.79 ± 0.47 at S_30_ to 0.71 ± 0.12 at S_6_ (Figure 3e). In this case, the ANOVA analysis showed highly significant differences between the mean values of different stations (*H* = 61.08, *p* = 0.0004525). The species richness values indicate more stable communities [12], which is inconsistent with other reports [45,46], possibly due to the differences in habitat as those studies were conducted in rivers. The highest species evenness value (0.59 ± 0.06) was found at S_6_ and the lowest (0.23 ± 0.03) was found at S_15_ (Figure 3f). Highly significant differences were detected at different stations (*H* = 57.04, *p* = 0.00142), as revealed by the ANOVA analysis. The species evenness ranged from 0.23 to 0.6 in the present study, which indicates a moderately stable algae community [11] in the homestead ponds, which is consistent with [20,47,48,49]. The Shannon—Wiener diversity varied from 2.23 ± 0.21 at S_19_ to 0.35 ± 0.05 at S_9_ (Figure 3g). The one-way ANOVA analysis showed highly significant differences in the mean diversity values at different stations (*H* = 65.04, *p* = 0.0001396). The Shannon–Wiener diversity values indicate a moderate diversity of algae [10] in homestead ponds which is similar to previous reports [20,44,47,49].

Legend: Ac—*Actinoptycus* spp.; Ap—*Aphanocapsa* sp.; As—*Asterococcus* sp.; Au—*Aulocodiscus* sp.; Bo—*Botryococcus* sp.; Chl –*Chlrococcus* sp.; Ch—*Chlorella* spp.; Cl—*Cladophora* sp.; Clo—*Cloasterium* spp.; Co—*Coelastrum* spp.; Cos—*Cosinodiscus* spp.; Eug—*Euglena* spp.; Gom—*Gomphospheria* spp.; He—*Heamatococcus* spp.; Le—*Lemena* sp.; Lep—*Lepocinclis* spp.; Mic—*Microcystis* spp.; Na—*Navicula* spp.; Ni—*Nitzschia* sp.; Oo—*Oocystis* spp.; Pe—*Pediastrum* sp.; Ph—*Phacus* spp.; Pho—*Phormidium*; Pl—*Planktospheria* sp.; Ri—*Rivularia* sp.; Rho—*Rhodomonus* spp.; Se—*Selenastrum* spp.; Ste—*Stephanodiscus* sp.; Tra—*Traclomonus* sp.

### 3.3. Assemblage of Algae

**Figure 4:**
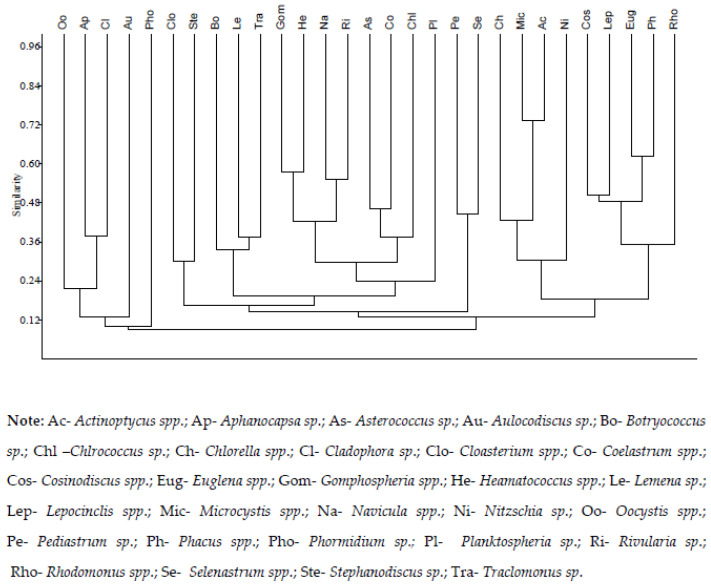
Cluster analysis based on Bray–Curtis similarity matrix of the most dominant 29 genera.

At 24% similarity, 11 major clusters were obtained from 29 dominant algae genera (Figure 4). The numbers of genera in each cluster were as follows: 4 algae genera (Oo, Au, Pho, Pl) remained isolated, 3 clusters contained 2 algal genera, 1 cluster contained 3 algal genera, 1 cluster contained 4 algal genera, 1 cluster contained 5 algal genera and last cluster contained 7 algae genera. Algal taxa with similar habitat preferences were grouped together.

### 3.4. Physico-Chemical Parameters

**Figure 5:**
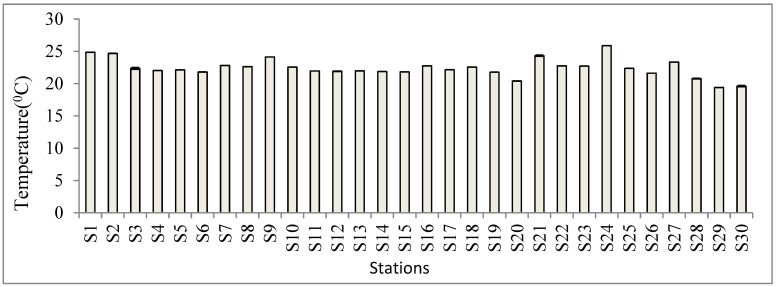

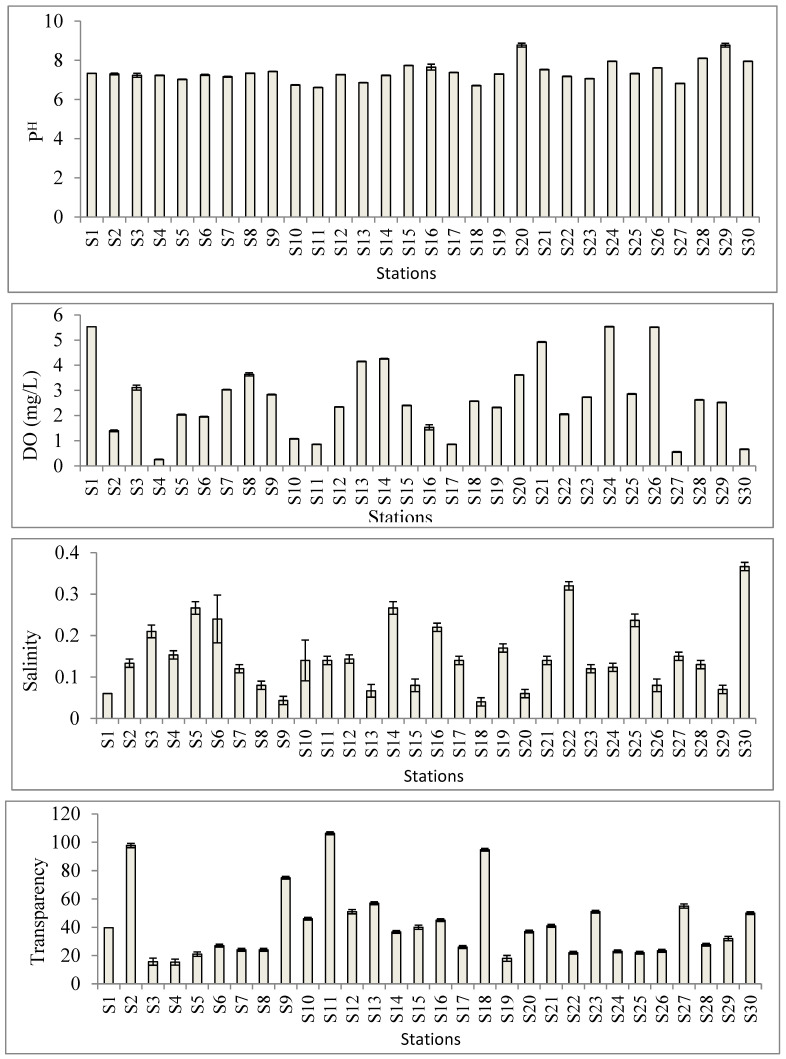

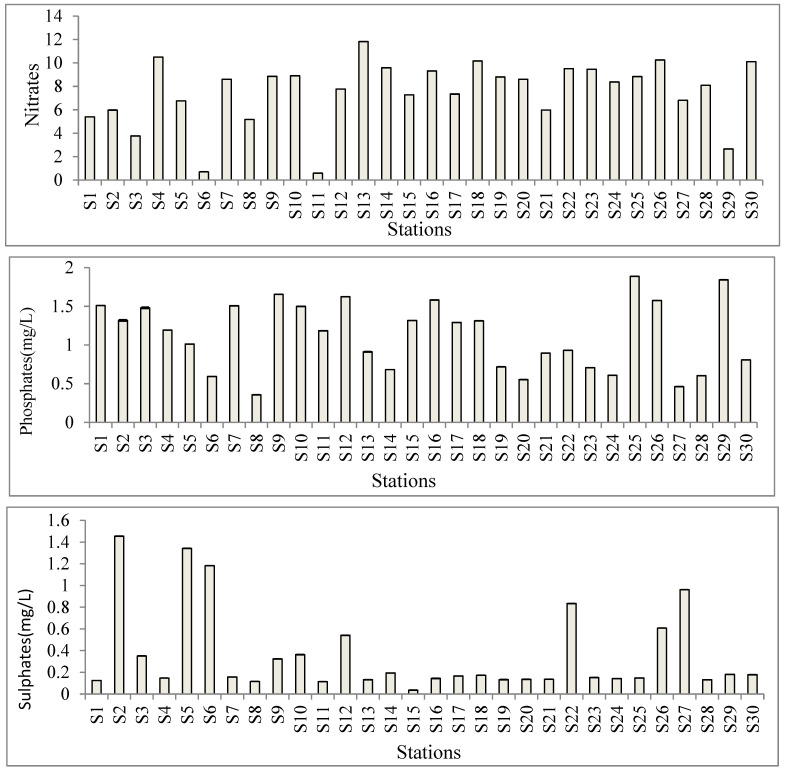
Physico-chemical variables at different stations/ponds.

The collected physico-chemical parameters of the homestead ponds in Noakhali are shown in Figure 5. A one-way ANOVA detected highly significant differences in the mean values of all measured environmental variables (*p* < 0.001). The physico-chemical parameters such as temperature, pH, DO and nutrients influence the distribution and abundance of algae and survival of aquatic organisms. The physico-chemical parameters are also the most important factor for any kind of growth, succession and variation in algae classes [50]. Highly significant differences were found in all of the physico-chemical parameters that were collected in this study. The temperature observations (19.6 ± 0.1 °C to 25.87 ± 0.01 °C) were very similar to previous reports [19,20,21,22,27,42,51]. The similar temperature observations at different stations may be due to the small size of homestead ponds [24]. In the present study, the pH values ranged from 6.61 ± 0.01 to 8.77 ± 0.01, which is a suitable range and consistent with [19,20,21,22,40,52]. Dissolved oxygen is one of the most critical parameters for survival of aquatic organisms [53]. Dissolved oxygen plays an important role for supporting aquatic life and is susceptible to environmental changes [54]. The range of DO was 0.56 ± 0.01 mg L^−1^ to 5.54 ± 0.02 mg L^−1^. The lower DO values may be due to changes in photosynthesis, respiration by fishes and other aquatic organisms, decomposition of organic materials, low water levels in winter or algal blooms. The salinity ranged from 0.04 ± 0.01 ppm to 0.37 ± 0.02 ppm in the study period. Although the ponds are rain-fed and supposed to be freshwater with zero salinity, the area is close to the sea, and seepage from underground might be a source of the salinity. The transparency ranged from 15.34 ± 1.53 cm to 106.34 ± 1.53 cm in the present study. Large differences and low values of transparency may be due to various human activities, geographical location, domestic sewage, low transportation of soil, sludge wash from adjoining areas and other organic matter through rain.

The concentration of nitrates varied from 0.59 ± 0.01 mg L^−1^ to 11.82 ± 0.01 mg L^−1^ during the present study. The relatively lower nitrates concentration was inconsistent with some other previous investigations [20,27,51,55], and may be the result of low or no fertilization and supplementary feed in the homestead ponds. High concentrations of nutrients such as nitrates, phosphates, and sulphates may result from surface run-off through heavy rainfall, and regeneration and release of nutrients from bottom sediments by increased turbulence and mixing [56]. The concentrations of phosphates were 0.36 ± 0.002 mg L^−1^ to 1.89 ± 0.001 mg L^−1^ in the present study, which was inconsistent with some other previous studies [20,27,51,57], and may be due to household activities that increase the phosphates originating from detergents and washing powders [58]. The concentration of sulphates varied from 0.04 ± 0.002 mg L^−1^ to 1.45 ± 0.002 mg L^−1^ in this study, which was inconsistent with earlier studies [20,38,55], possibly due to household activities, and the use of detergents and washing powders [58]. In this study, the sulphates concentration reached a minimum in winter seasons that agrees with [20,38,40,59].

### 3.5. Relationship between Physico-Chemical Parameters and Biological Variables

**Figure 6:**
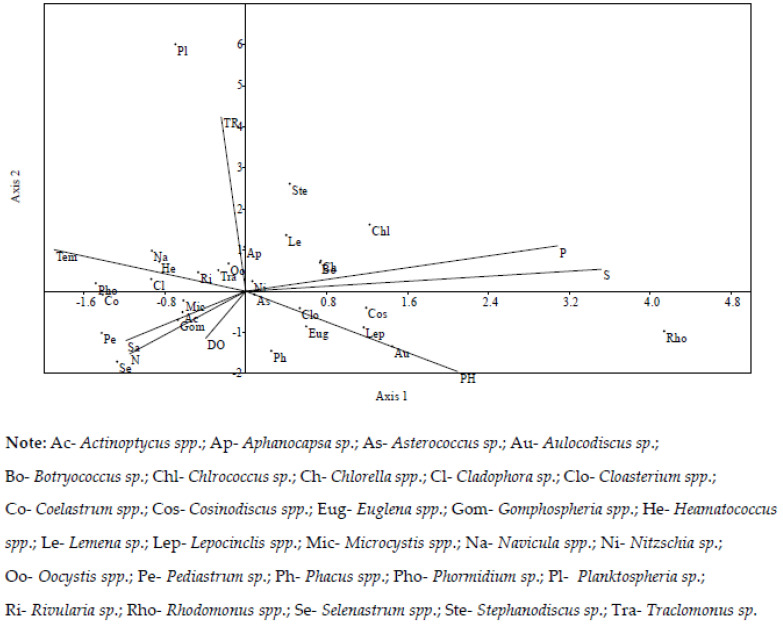
CCA bi-plot between 30 dominant algae genera and 8 physico-chemical parameters.

Canonical correspondence analysis (CCA) revealed a relationship between the physico-chemical parameters and the dominant algae genera. The CCA was plotted for 8 physico-chemical parameters and 29 dominant algae genera (Figure 6). The eigen value of axis 1 (0.19) indicates 43.69% correlation and that of axis 2 (0.1) indicates a 23.16% correlation between the physico-chemical parameters and dominant genera of algae. The DO, salinity, transparency, nitrates, phosphates and sulphates demonstrated maximum impact on the abundance of algae genera, whereas temperature, pH, transparency had medium effect on the abundance of algae communities. The abundance of *Traclomonus *sp., *Asterococcus *sp., *Oocystis* spp., and *Nitzschia* spp. were highly correlated with the physico-chemical parameters, while *Actinoptycus* spp., *Rivularia* sp., *Microcystis* spp., *Gomphospheria* spp., *Aphanocapsa* sp., *Lemena* sp., *Chlorella* spp., *Botryococcus* sp., *Cloasterium* spp., *Euglena* spp., *Phacus *spp. were moderately correlated with physico-chemical parameters. Phosphates and sulphates were positively correlated with *Aphanocapsa* sp., *Nitzschia* spp., *Lemena* sp., *Stephanodiscus* sp., *Chlorella* spp., *Botryococcus* sp., *Chlrococcus* sp., and with both axes. *Aphanocapsa* sp., *Nitzschia* spp., *Lemena* sp., *Stephanodiscus* sp., *Chlorella* spp., *Botryococcus* sp., and *Chlrococcus* sp., were positively correlated with both axes. *Microcystis* spp., *Actinoptycus* spp., *Gomphospheria* spp., *Pediastrum* sp., and *Selenastrum* spp. were negatively correlated with both axes and with salinity, nitrates and dissolved oxygen. *Oocystis* spp., *Traclomonus* sp., *Rivularia* sp., *Heamatococcus* spp., *Navicula* spp., *Cladophora* sp., and *Phormidium* sp., had a positive relationship with temperature, transparency and were positively correlated with axis 2 and negative with axis 1. *Asterococcus* sp., *Phacus* spp., *Cloasterium* spp., *Euglena* spp, *Cosinodiscus* spp., *Lepocinclis* spp., *Aulocodiscus* sp., and *Rhodomonus* spp., had a positive relationship with pH and showed a positive relationship with axis 1 and negative relationship with axis 2.

The Pearson’s correlation coefficient among the physico-chemical parameters, abundance of algae classes and diversity indices in the homestead ponds are presented in Table 2. The abundance of Bacillariophyceae has a positive correlation with sulphates (r = 0.47, *p* < 0.05). Chlorophyceae has a positive correlation with phosphates (r = 0.37, *p* < 0.05), sulphates (r = 0.41, *p* < 0.05) and Bacillariophyceae (r = 0.56, *p* < 0.01). Chrysophyceae has negative relationship with temperature (r = −0.38, *p* < 0.05) and has a positive correlation with pH (r = 0.45, *p* < 0.05), phosphates (r = 0.42, *p* < 0.05) and sulphates (r = 0.46, *p* < 0.05). Euglenophyceae has a positive correlation with phosphates (r = 0.47, *p* < 0.05), Chlorophyceae (r = 0.43, *p* < 0.05) and Chrysophyceae (r = 0.43, *p* < 0.05). Rhodophyceae has a negative correlation with Cyanophyceae (r = −0.51, *p* < 0.01). The abundance of total algae has a positive correlation with phosphates (r = 0.41, *p* < 0.05), sulphates (r = 0.52, *p* < 0.01), Bacillariophyceae (r = 0.60, *p* < 0.01), Chlorophyceae (r = 0.78, *p* < 0.01), Chrysophyceae (r = 0.40, *p* < 0.05) and Euglenophyceae (r = 0.64, *p* < 0.01). The Shannon–Wiener diversity index has a negative correlation with phosphates (r = −0.41, *p* < 0.05) and sulphates (r = −0.043, *p* < 0.05). Species richness has negative correlation with Cyanophyceae (r = −0.41, *p* < 0.05). The dominance index has a negative relationship with Bacillariophyceae (r = −0.47, *p* < 0.05), Chlorophyceae (r = −0.62, *p* < 0.01), species evenness (r = −0.66, *p* < 0.01) and species richness (r = −0.38, *p* < 0.05).

In this study, physico-chemical parameters such as temperature, transparency, pH and nutrients (nitrates, phosphates, sulphates) have maximum impact on the abundance of harmful algae genera. Previous studies also reported that temperature, transparency, dissolved oxygen, nitrates, sulphates and phosphates are the main factors determining algae genera [9,20,60]. Physico-chemical parameters such as nutrients and temperature may affect the algae abundance [61]. Pearson’s correlation showed that the total algal abundance has a strong positive correlation with sulphates which was also supported by earlier studies [20,38]. Some algae classes such as Bacillariophyceae, Chlorophyceae, and Chrysophyceae were positively correlated with sulphates. Chlorophyceae and Chrysophyceae were also positively correlated with phosphates. Chrysophyceae was positively correlated with pH and negatively correlated with temperature.

## 4. Conclusions

The physico-chemical characteristics, abundance, diversity, and community structure of harmful algae from homestead ponds along the central coast of Bangladesh are all summarized for the first time in this study. A total of 89 genera of algae from 10 classes were reported, and of these, 81 genera belonging to 7 classes, were identified as harmful algae. The class Chlorophycea, which had 32 genera, contained the most genera from these harmful classes, while Cyanophyceae was the leading class with a 50.81% contribution. Among the 29 prevalent genera of algae, 28 belonged to harmful groups, of which *Microcystis* spp. alone was responsible for 28.24% of total abundance. At a level of 24% similarity, cluster analyses revealed 11 significant clusters among the dominant algal taxa. The total algae abundance was mainly governed by phosphates and sulphates (according to Pearson’s correlation). According to the CCA analysis, DO, salinity, transparency, nitrates, phosphates, and sulphates have the maximum impact on the abundance of algae genera. The present study contributes to the basic knowledge on physico-chemical parameters, diversity, abundance and influence of environmental drivers on harmful algae in homestead fish ponds in a tropical coastal area. The results can be utilised for further study by researchers, farmers and policy makers to identify and control these toxic algal groups.

## Figures and Tables

**Table 1:** List of Algae genera recorded from the homestead ponds of coastal Noakhali.

Algae Class	Name of Algae Genera
* Bacillariophyceae (20)	*Chaetoceros* sp., *Cocconies* sp., *Cosinodiscus* spp., *Cosmarium* sp., *Cyclotella* sp., *Diatom* spp., *Ellerbackia* sp., *Epithemia* sp., *Fragilaria* sp., *Gomphonema* sp., *Gyrosigma* sp., *Hemidiscus* sp., *Hyalodiscus* sp., *Meridion* sp., *Navicula* spp., *Nitzschia* sp., *Peronia* sp., *Tabellaria* sp., *Triceratium* sp., *Rhoicosphenia* sp.
* Chlorophyceae (32)	*Actinastrum* sp., *Ankristodesmus* sp., *Asterococcus* sp., *Botryococcus* sp., *Bulbocheate* sp., *Chlamydomonus* sp., *Chorella* spp., *Cladophora* sp., *Closterium* spp., *Coelestrum* spp., *Crucigenia* sp., *Draparnaldia* sp., *Enteromorpha* sp., *Eudorina* sp., *Gonium* sp., *Heamatococcus* spp., *Hydrodictyon* sp., *Mesotaenium* sp., *Oocystis* spp., *Pediastrum* sp., *Planktosphaera* sp., *Pleurococcus* sp., *Pleurotaenium* sp., *Scenedesmus* spp., *Selenastrum* spp., *Spirogyra* sp., *Staurastrum* sp., *Tetradron* sp., *Tetraspora* sp., *Volvox* sp., *Xanthidium* sp., *Zygnema* sp.
* Chrysophyceae (2)	*Mallomonas* sp., *Uroglena* sp.
Cosinodiscophyceae (2)	*Cerataulina* sp., *Stephanodiscus* sp.
* Cryptophyceae (1)	*Rhodomonus* sp.
* Cyanophyceae (21)	*Actinoptychus* spp., *Amphiprora* sp., *Anabaena* sp, *Aphanizomenon* sp., *Aphanocapsa* sp., *Aphanothece* sp., *Arthrospira* sp., *Aulacodiscus* sp., *Chlrococcus* sp., *Coelosphaerium* spp., *Gloecapsa* spp., *Gloeotrichia* sp., *Gomphosphaeria* spp., *Microcystis* spp., *Nostoc* sp., *Oscillatoria* sp., *Phormidium* sp., *Pseudanabena* sp., *Rivularia* spp., *Stigonema* sp., *Tolypothrix* sp.
* Euglenophyceae (4)	*Euglena* spp., *Lepocinclis* spp., *Phacus* spp., *Tracehlomonas* sp.
Fragilariophyceae (2)	*Asterionella* sp., *Thalassiothrix* sp.
* Rhodophyceae (1)	*Lemanea* sp.
Zygnemophyceae (4)	*Actinotaenium* sp., *Desmidium* sp., *Micrasterias* sp., *Mougeotia* sp.

Note: “*” Harmful algae classes.

**Table 2:** Pearson’s correlation coefficients for physico-chemical parameters, algae abundance and diversity indices.

0	Tem	PH	DO	Sa	TR	N	P	S	BAC	CHL	CRY	CYA	EUG	RH	TA	H’	J’	d	D
Tem	0.00																		
PH	−0.38	0.00																	
DO	0.33	0.24	0.00																
Sa	−0.22	−0.10	−0.35	0.00															
TR	0.17	−0.34	−0.23	−0.29	0.00														
N	−0.02	−0.08	0.14	−0.03	−0.06	0.00													
P	0.01	−0.04	−0.05	−0.17	0.12	−0.03	0.00												
S	−0.24	0.02	−0.17	−0.10	0.05	0.17	0.75	0.00											
BAC	−0.04	−0.04	−0.22	−0.04	−0.05	0.20	0.35	**0.47**	0.00										
CHL	0.07	−0.10	−0.05	−0.04	0.19	−0.15 *	**0.37**	**0.41**	***0.56***	0.00									
CRY	**−0.38**	**0.45**	−0.04	−0.07	−0.17	−0.24	**0.42**	**0.46**	0.25	0.28	0.00								
CYA	0.22	−0.03	−0.03	0.31	−0.37	−0.20	−0.27	−0.31	−0.26	−0.16	−0.26	0.00							
EUG	−0.15	0.08	−0.09	0.08	−0.35	0.15	0.35	**0.47**	0.35	**0.43**	**0.43**	−0.19	0.00						
RH	−0.10	−0.05	0.22	−0.29	0.22	0.15	0.24	0.22	0.35	0.10	0.35	***−0.51***	−0.07	0.00					
TA	−0.05	0.06	−0.18	−0.02	−0.14	−0.12	**0.41**	***0.52***	***0.60***	***0.78***	**0.40**	−0.05	***0.64***	0.00	0.00				
H’	−0.21	0.04	−0.08	0.25	−0.32	−0.22	**−0.41**	**−0.43**	−0.03	0.06	−0.04	0.17	−0.10	−0.05	0.04	0.00			
J’	0.01	−0.09	−0.17	0.06	−0.02	−0.21	−0.10	−0.03	0.19	0.36	0.04	0.20	0.29	0.00	0.20	0.23	0.00		
d	−0.36	0.21	0.02	0.07	0.09	0.30	−0.02	0.11	0.22	0.16	0.28	**−0.41**	0.04	0.36	0.09	0.12	−0.35	0.00	
D	0.19	−0.04	0.19	−0.12	−0.03	0.03	0.00	−0.13	**−0.47**	***−0.62***	−0.31	0.06	−0.31	−0.18	−0.36	−0.34	***−0.66***	**−0.38**	0.00

**Note:** Tem—Temperature, DO—Dissolved oxygen, Sa—Salinity, TR—Transparency, N—Nitrates, P—Phosphates, S—Sulphate, BAC—Bacillariophyceae, CHL—Chlorophyceae, CRY—Chrysophyceae, CYA—Cyanophyceae, EUG—Euglenophyceae, RH—Rhodophyceae, TA—Total algae, H′—Shannon–Wiener diversity index, J′—Species evenness, d—species richness, D—dominance index. Bold—Significant correlation coefficient, “only bold”—significant at *p* < 0.05, “bold + italic”—significant at *p* < 0.05.

## Data Availability

Data are provided in the article.

## References

[1] Wickstead J.H. (1965). Introduction to the Study of Tropical Plankton.

[2] Suseela M.R., Anand N. (2009). Conservation and Diversity of Fresh Water Algae. Biology and Biodiversity of Microalgae.

[3] Telesh I.V. (2004). Plankton of the Baltic estuarine ecosystem with emphasis on Neva estuary: A review of present knowledge and research perspectives. Mar. Pollut. Bull..

[4] Yakubu A.F. (2000). A comparative study of phytoplankton communities of some rivers, creeks and burrow pits in the Niger Delta area. J. Appl. Sci. Environ. Manag..

[5] Nuccio C., Melillo C., Massi L., Innamorati M. (2003). Phytoplankton abundance, community structure and diversity in the eutrophicated Orbetello lagoon (Tuscany) from 1995 to 2001. Oceanol. Acta.

[6] Song X., Huang L., Zhang J., Huang X., Zhang J., Yin J., Tan Y., Liu S. (2004). Variation of phytoplankton biomass and primary production in Daya Bay during spring and summer. Mar. Pollut. Bull..

[7] Bahaar S.W.N., Bhat G.A. (2011). Aquatic bio diversity in the paddy fields of Kashmir valley (J and K), India. Asian J. Agric. Res..

[8] Margalef R. (1978). Life forms of phytoplankton as survival alternatives in an unstable environment. Oceanol. Acta.

[9] Al M.A., Akhtar A., Hassan M.L., Rahman M.F., Warren A. (2019). An approach to analyzing environmental drivers of phytoplankton community patterns in coastal waters in the northern Bay of Bengal, Bangladesh. Reg. Stud. Mar. Sci..

[10] Odum E.P., Barrett G.W. (1971). Fundamentals of Ecology.

[11] Krebs C.H.J. (1989). Ecological Methodology.

[12] Margalef R. (1968). Perspectives in Ecological Theory.

[13] Reynolds C.S. (1987). The responsible of phytoplankton communities to changing lake environments. Swiss J. Hydrol..

[14] Chaturvedi R.K., Sharma K.P., Sharma K., Bhardwaj S.M., Sharma S. (1999). Plankton community of polluted waters around Sanganer, Jaipur. J. Environ. Pollut..

[15] Saravanakumar A., Rajkumar M., Thivakaran G.A., Serebiah J.S. (2008). Abundance and seasonal variations of phytoplankton in the creek waters of western mangrove of Kachchh-Gujarat. J. Environ. Biol..

[16] Hossain M.Y., Begum M., Ahmed Z.F., Hoque M.A., Karim M.A., Wahab M.A. (2006). A study on the effects of iso- phosphorus fertilizers on plankton production in fish ponds. South Pac. Stud..

[17] Belton B., Azad A. (2012). The characteristics and status of pond aquaculture in Bangladesh. Aquaculture.

[18] E-Jahan K.M., Ahmed M., Belton B. (2010). The impacts of aquaculture development on food security: Lessons from Bangladesh. Aquac. Res..

[19] Akter S., Rahman M.M., Faruk A., Bhuiyan M.N.M., Hossain A., Asif A.A. (2018). Qualitative and quantitative analysis of phytoplankton in culture pond of Noakhali district, Bangladesh. Int. J. Fish. Aquat. Stud..

[20] Sarker M.M., Hossain M.B., Islam M.M., Kamal A.H.M., Idris M.H. (2020). Unravelling the diversity and assemblage of phytoplankton in homestead ponds of central coastal belt, Bangladesh. Aquac. Res..

[21] Khan N.S., Bari J.B.A. (2019). The effects of physico-chemical parameters on plankton distribution in poultry manure and artificial formulated feed treated fish ponds, Noakhali, Bangladesh. Int. J. Fish. Aquat. Stud..

[22] Khan N.S., Uddin A., Bari J.B.A., Tisha N.A. (2019). Evaluation the potentiality of ancient ponds by palmer’s Algal pollution index, Noakhali, Bangladesh. Int. J. Fish. Aquat. Res..

[23] Ahmed S., Rahman A.F.M.A., Hossain M.B. (2013). Phytoplankton biodiversity in seasonal waterlogged paddy fields, Bangladesh. Ecologia.

[24] Hossain M.Y., Jasmine S., Ibrahim A.H.M., Ahmed Z.F., Ohtomi J., Fulanda B., Wahab M.A. (2007). A preliminary observation on water quality and plankton of an earthen fish pond in Bangladesh: Recommendations for future studies. Pak. J. Biol. Sci..

[25] Begum M., Hossain M.Y., Wahab M.A., Ahmed Z.F., Alam M.J., Shah M.M.R., Jasmine S. (2007). Effects of iso- nutrient fertilization on plankton production in earthen ponds of Bangladesh. Pak. J. Biol. Sci..

[26] Chowdhury A.H., Al Mamun A. (2006). Physio-chemical conditions and plankton population of two fishponds in Khulna. Univ. J. Zool. Rajshahi Univ..

[27] Affan A., Jewel A.S., Haque M., Khan S., Lee J.B. (2005). Seasonal cycle of phytoplankton in aquaculture ponds in Bangladesh. ALGAE.

[28] Bellinger E.G., Sigee D.C. (2015). A Key to the More Frequently Occurring Freshwater Algae. Freshwater Algae: Identification, Enumeration and Use as Bioindicators.

[29] Belcher H., Swale E. (1976). A Beginner’s Guide to Freshwater Algae.

[30] Davis C.C. (1955). The Marine and Fresh-Water Plankton.

[31] Manickam N., Bhavan P.S., Vijayan P., Sumathi G. (2012). Phytoplankton species diversity in the parambikulam-aliyar irrigational canals (Tamil Nadu, India). Int. J. Pharma Bio Sci..

[32] Manickam N., Bhavan P.S., Santhanam P., Muralisankar T., Kumar S.D., Balakrishnan S., Ananth S., Devi A.S. (2020). Phytoplankton bio-diversity in the two perennial lakes of Coimbatore, Tamil Nadu, India. Acta Ecol. Sin..

[33] Pitchaikani J.S., Lipton A.P. (2016). Nutrients and phytoplankton dynamics in the fishing grounds off Tiruchendur coastal waters, Gulf of Mannar, India. SpringerPlus.

[34] Pielou E.C. (1966). Species-diversity and pattern-diversity in the study of ecological succession. J. Theor. Biol..

[35] Shannon C.E., Weaver W. (1949). Mathematical Theory of Communication.

[36] APHA (1992). Standard Methods for Examination of Water and Waste Water.

[37] Hammer Ø., Harper D.A., Ryan P.D. (2001). PAST: Paleontological statistics Software package for education and data analysis. Palaeontol. Electron..

[38] Sharma R.C., Tiwari V. (2018). Phytoplankton diversity in relation to physico-chemical environmental variables of Nachiketa Tal, Garhwal Himalaya. Biodivers. Int. J..

[39] Shah M.M.R., Hossain M.Y., Begum M., Ahmed Z.F., Ohtomi J., Rahman M.M., Alam M.J., Islam M.A., Fulanda B. (2008). Seasonal variations of phytoplankton community structure and production in relation to environmental factors of the southwest coastal waters of Bangladesh. J. Fish. Aquat. Sci..

[40] Gogoi P., Sinha A., Sarkar S.D., Chanu T.N., Yadav A.K., Koushlesh S.K., Borah S., Das S.K., Das B.K. (2019). Seanoal influence of physicochemical parameters on phytoplankton diversity and assemblage pattern in Kailash Khal, a tropical wetland, Sundarbans, India. Appl. Water Sci..

[41] Iqbal M.M., Billah M.M., Haider M.N., Islam M.S., Payel H.R., Bhuiyan M.K.A., Dawood M.A.O. (2017). Seasonal distribution of phytoplankton community in a subtrophical estuary of the south-eastern coast Bangladesh. Zool. Ecol..

[42] Hossain M.I., Alam M.M., Kamal B.M.M., Galib S.M. (2013). Investigation of phytoplankton and physico-chemical parameters in nursery, Growout and Broodstock Ponds. J. Sci. Res..

[43] Chowdhury M.M.R., Mondol M.R.K., Sarker C. (2007). Seasonal variation of plankton population of Borobila beel in Rangpur district. Univ. J. Zool. Rajshahi Univ..

[44] Effendi H., Kawaroe M., Lestari D.F., Mursalin, Permadi T. (2016). Distribution of phytoplankton diversity and abundance in Mahakam Delta, East Kalimantan. Procedia Environ. Sci..

[45] Hossain M.R.A., Pramanik M.M., Hasan M.M. (2017). Diversity indices of plankton communities in the River Meghna of Bangladesh. Int. J. Fish. Aquat. Stud..

[46] Ahsan D.A., Kabir A.N., Rahman M.M., Mahabub S., Yesmin R., Faruque M.H., Naser M.N. (2012). Plankton composition, abundance and diversity in hilsa (Tenualosa ilisha) migratory rivers of Bangladesh during spawning season. Dhaka Univ. J. Biol. Sci..

[47] Sidik M.J., Rashed-Un-Nabi M., Hoque M.A. (2008). Distribution of phytoplankton community in relation to environmental parameters in cage culture area of Sepanggar Bay, Sabah, Malaysia. Estuar. Coast. Shelf Sci..

[48] Gao X.L., Song J.M. (2005). Phytoplankton distribution and their relationship with the environment in the Changjiang Estuary, China. Mar. Pollut. Bull..

[49] Huang L., Jian W., Song X., Huang X., Liu S., Qian P., Yin K., Wu M. (2004). Species diversity and distribution for phytoplankton of the Pearl River estuary during rainy and dry seasons. Mar. Pollut. Bull..

[50] Aktan Y., Tufekci V., Tufekci H., Aykulu G. (2005). Distribution patterns, biomass estimates and diversity of phytoplankton in Izmit Bay (Turkey). Estuar. Coast. Shelf Sci..

[51] Haider M.A., Shahriar S.I.M., Hosen M.H.A., Chhanda M.S., Khatun M.M. (2017). A study on water quality parameters and benthos abundance in freshwater homestead ponds of Dinajpur, Bangladesh. Int. J. Fish. Aquat. Stud..

[52] Munni M.A., Fardus Z., Mia M.Y., Afrin R. (2013). Assessment of pond water quality for fish culture: A case study of Santosh region in Tangail, Bangladesh. J. Environ. Sci. Nat. Resour..

[53] Sharma R.C., Singh N., Chauhan A. (2016). The influence of physico-chemical parameters on phytoplankton distribution in a head water stream of Garhwal Himalayas: A case study. Egypt. J. Aquat. Res..

[54] Ahmed K.K.U., Ahamed S.U., Hossain M.R.A., Ahmed T., Barman S. (2003). Quantitative and qualitative assessment of plankton: Some ecological aspect and water quality parameters of river Meghna, Bangladesh. Bangladesh J. Fish. Res..

[55] Hossain M.A.R. (2014). An overview of fisheries sector of Bangladesh. Res. Agric. Livest. Fish..

[56] Thillai R.K., Rajkumar M., Sun J., Ashok P.V., Perumal P. (2010). Seasonal variations of phytoplankton diversity in the Coleroon coastal waters, southeast coast of India. Acta Oceanol. Sin..

[57] Sultana T., Saifullah A.S.M., Moniruzzaman M., Rpy S., Al Mamun S. (2012). Assessment of surface water quality: A case study of Tangail Municiple area, Bangladesh. Bangladesh J. Sci. Res..

[58] Gambhir R.S., Kapoor V., Nirola A., Sohi R., Bansal V. (2012). Water pollution: Impact of pollutants and new promising techniques in purification process. J. Hum. Ecol..

[59] Hossain M.S., Uddin M.J., Fakhruddin A.N.M. (2013). Impacts of shrimp farming on the coastal environment of Bangladesh and approach for management. Rev. Environ. Sci. Bio/Technol..

[60] Zebek E. (2015). Response of planktonic cyanobacteria and periphyton assemblages to physicochemical properties of stormwater in a shallow urban lake. J. Elem..

[61] Macedo M.F., Duarte P., Mendes P., Ferreira J.G. (2001). Annual variation of environmental variables, phytoplankton species composition and photosynthetic parameters in a coastal lagoon. J. Plankton Res..

